# Application of CRISPR-Cas9-Based Gene Editing Technology in Inherited Liver Diseases

**DOI:** 10.3390/ijms27146469

**Published:** 2026-07-21

**Authors:** Ran Liu, Shiqi Cong, Yuan Gao, Jiaqi Xu, Xiaoxia Shi

**Affiliations:** Liaoning Provincial Key Laboratory of Biotechnology and Molecular Drug Research and Development, School of Life Sciences, Liaoning Normal University, Dalian 116082, China; august_liu9@163.com (R.L.);

**Keywords:** gene editing, CRISPR-Cas9, inherited liver disease

## Abstract

Inherited liver diseases are predominantly caused by monogenic mutations, and the vast majority of these conditions currently lack curative treatment options. Although liver transplantation may be used for patients with end-stage disease, it faces numerous challenges, including donor organ shortage, immune rejection, and the need for lifelong immunosuppression. In recent years, CRISPR-Cas9-based gene editing technology has advanced rapidly, offering transformative hope for the treatment of these diseases. This review systematically elucidates the working principles and technical advantages of the CRISPR-Cas9 system and its derived tools (base editing and prime editing), summarizes recent applications of these technologies in the treatment of hereditary liver diseases, and discusses the prospects and challenges of their clinical translation, aiming to provide a theoretical reference for future research in this field.

## 1. Introduction

The liver is the largest parenchymal organ, and it handles metabolism, synthesis, and excretion. It produces bile salts to aid fat and vitamin absorption, detoxifies compounds through bile, synthesizes clotting factors and albumin, and helps manage blood lipids and glucose levels [1]. Mutations in related genes can disrupt these roles, leading to liver dysfunction and secondary damage to the blood, kidneys, or brain. Diseases caused by such gene mutations in hepatocytes are collectively referred to as inherited liver diseases, examples of which include hereditary tyrosinemia type 1 (HT1), Crigler–Najjar syndrome (CNS), and progressive familial intrahepatic cholestasis (PFICs) [2]. Currently, apart from liver transplantation, there is a lack of fundamentally effective treatments for most inherited liver diseases. However, considering the scarcity of liver donors, the high risk and cost of transplantation, and the long-term immune rejection after surgery, there is an urgent need to develop new effective and safe therapeutic approaches.

The emergence of gene editing therapy offers new hope and ideas for the treatment of inherited liver diseases. Among various gene editing tools, CRISPR-Cas9 technology is one of the most promising due to its high editing efficiency, ease of design, and broad applicability. In recent years, with the continuous optimization of delivery systems and editing tools—including the development of novel editing systems such as base editing and prime editing based on the CRISPR-Cas9 framework—the safety of gene editing has been significantly improved and the risk of off-target effects reduced. These advances have led to remarkable progress in the treatment of inherited liver diseases, with multiple studies successfully validating the therapeutic potential of these tools in animal models and even in clinical trials.

In this paper, we systematically review the fundamental principles, delivery strategies, and research progress of CRISPR-Cas9, base editing, and prime editing technologies in the treatment of inherited liver diseases; analyze current challenges and countermeasures; and discuss future development directions, with the goal of providing a reference for research in related fields.

## 2. CRISPR-Cas-Mediated Gene Editing

### 2.1. Structure and Mechanism of CRISPR-Cas9

**Figure 1:**
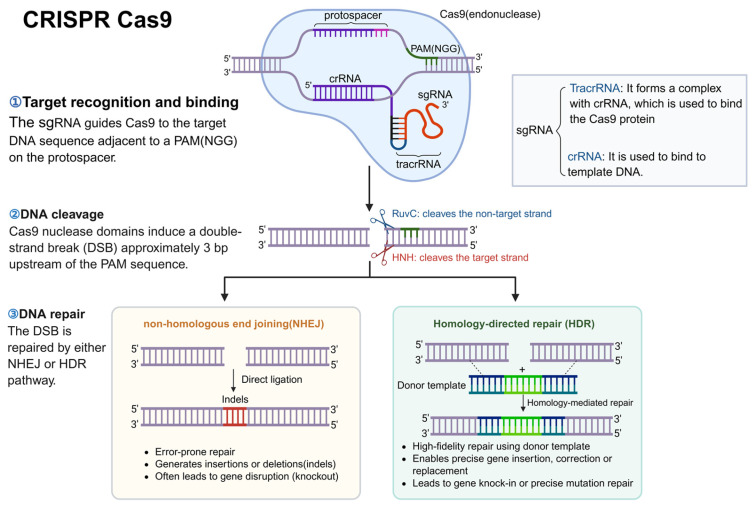
Mechanism of CRISPR-Cas9-mediated gene editing. The sgRNA, composed of crRNA and tracrRNA, guides Cas9 to a protospacer adjacent to an NGG PAM. The HNH and RuvC domains cleave the target and non-target strands, respectively, approximately 3 bp upstream of the PAM to generate a double-strand break. NHEJ directly rejoins the broken ends and commonly produces indels leading to gene knockout, whereas HDR uses a donor template for gene insertion, correction, or replacement.

CRISPR-Cas9 is composed of the Cas9 protein and a single-guide RNA (sgRNA). The Cas9 protein acts as a pair of molecular scissors with nuclease activity, while the sgRNA consists of tracrRNA and crRNA. The tracrRNA hybridizes with the crRNA to form a complex that associates with the Cas9 protein, and the crRNA can bind to the target DNA strand [3]. The target DNA sequence is termed the protospacer and is located adjacent to a protospacer adjacent motif (PAM; 5′-NGG-3′ for SpCas9), which is required for target recognition. As shown in Figure 1, the sgRNA binds to the target DNA sequence, after which the HNH and RuvC nuclease domains of Cas9 cleave the target and non-target strands, respectively, approximately 3 bp upstream of the PAM, resulting in a double-strand break (DSB) [3,4,5]. In cells, broken DNA is primarily repaired through one of two major repair pathways: non-homologous end joining (NHEJ) or homology-directed repair (HDR), generating a new DNA strand [6]. CRISPR-Cas9 technology enables both gene knockout and knock-in. Typically, after a DSB occurs, cells activate self-repair mechanisms via NHEJ, which directly ligates the broken ends but often introduces small insertions or deletions (indels) that disrupt the gene, thereby achieving gene knockout [7]. To achieve knock-in of a functional gene, an exogenous DNA donor template is supplied, and cells may perform HDR, which inserts the functional gene at the target locus, resulting in successful gene knock-in [7]. However, Cas9-induced DSBs may also produce unintended indels and off-target changes [8].

### 2.2. Progress of CRISPR-Cas9 in the Treatment of Inherited Liver Diseases

Since its inception, CRISPR-Cas9 technology has rapidly become an important tool for research on the treatment of inherited liver diseases due to its high genome-editing capability [9,10,11]. Although its reliance on DNA double-strand breaks and endogenous repair mechanisms presents certain limitations, numerous preclinical studies have validated its therapeutic potential in various hereditary liver disease models and have laid a critical foundation for the subsequent development of gene-editing technologies.

Hereditary tyrosinemia type 1 (HT1): In 2014, Anderson et al. used the CRISPR-Cas9 system to achieve in vivo correction of the fumarylacetoacetate hydrolase (*FAH*) gene mutation in an adult mouse model of hereditary tyrosinemia via hydrodynamic tail vein injection. The initial editing efficiency was approximately 0.40%; however, because the corrected hepatocytes possessed a significant positive selection advantage, their proportion expanded to 33.5% after proliferation within the liver, successfully rescuing the phenotypes of body weight loss and liver injury [12]. This study provided an important proof-of-concept for CRISPR-mediated gene therapy of an adult genetic disease. It revealed the key role of the selective proliferative advantage of corrected hepatocytes in achieving therapeutic benefits even at a low initial editing efficiency. However, this selective expansion also raises a potential safety concern. Although the study did not demonstrate the preferential expansion of off-target-edited cells, rare hepatocytes carrying unintended edits could, in principle, undergo clonal enrichment if such alterations confer a survival or proliferative advantage. Therefore, the increase in FAH-positive hepatocytes from approximately 0.4% to 33.5% should be interpreted not only as evidence of therapeutic efficacy but also as highlighting the need for sensitive genome-wide off-target assessment, clonal lineage tracing, and long-term monitoring for abnormal clonal expansion and tumorigenic risk.

Ornithine transcarbamylase deficiency (OTCD): In 2016, Yang, Y. et al. [13] used a dual adeno-associated virus (AAV8) vector system to deliver SaCas9 and a targeting sgRNA, systematically evaluating the impact of treatment timing on efficacy and safety in a neonatal mouse model of ornithine transcarbamylase deficiency (OTCD). Neonatal treatment effectively restored approximately 10% of allele function, significantly improving survival and blood ammonia levels under a high-protein diet. In a separate cohort of adult mice, however, adult mice showed low gene correction efficiency accompanied by large deletions, leading to further loss of residual ornithine transcarbamylase (OTC) function and severe hyperammonemia. This study was the first one that clearly demonstrated that treatment timing critically influences the efficacy and safety of CRISPR-Cas9 gene therapy, providing important guidance for intervention strategies in neonatal inherited liver diseases [13].

Alpha-1 antitrypsin deficiency (AATD): In 2018, Song, C.Q. et al. [14] used a dual-AAV vector to deliver a CRISPR-Cas9 system for in vivo gene editing of the Z-AAT mutation associated with alpha-1 antitrypsin deficiency in a mouse model. The results showed that the system partially restored serum M-AAT levels (45 μg/mL in neonatal mice and 71 μg/mL in adult mice) and achieved approximately 1.9% precise gene correction. Although this level did not reach the full therapeutic threshold, the study provided an important proof-of-concept for CRISPR-mediated AATD gene therapy, and no obvious off-target effects were detected [14].

Familial hypercholesterolemia (FH): In 2020, Zhao, H. et al. [15] used an AAV8-delivered CRISPR-Cas9 system to perform neonatal hepatocyte gene editing in a mouse model of familial hypercholesterolemia. By precisely correcting the mutation via the HDR mechanism, they achieved approximately 6.7% allele correction efficiency, restored low-density lipoprotein receptor (LDLR) protein expression in about 20% of hepatocytes, and reached LDLR protein levels equivalent to 18% of wild-type controls. The treatment significantly reduced serum total cholesterol, triglycerides, and low-density lipoprotein cholesterol (LDL-C) levels, effectively alleviated atherosclerotic plaque area and hepatic lipid accumulation, and no obvious hepatotoxicity or off-target effects were observed. Furthermore, in subsequent adult-stage assessments, a partial recovery of the LDLR protein and a significant improvement in the atherosclerotic phenotype were still observed. This study demonstrated the feasibility of achieving long-term amelioration of metabolic liver disease through HDR-mediated editing in the neonatal period [15].

In addition to viral vectors, Chen et al. investigated whether lipid nanoparticles (LNPs) could provide transient, non-viral delivery of CRISPR ribonucleoproteins (RNPs) for in vivo genome editing. The study had two main objectives: to improve the activity and PAM compatibility of the thermostable Geobacillus stearothermophilus Cas9 (GeoCas9) by directed evolution, generating iGeoCas9, and to evaluate tissue-selective LNP formulations for delivery of iGeoCas9-sgRNA RNPs. Delivery and editing efficiency were first assessed in Ai9 tdTomato reporter mice. These animals are not a disease model; they carry a tdTomato reporter silenced by a loxP-flanked SV40 poly(A) STOP cassette, and CRISPR-mediated disruption of this cassette activates tdTomato fluorescence, allowing edited cells to be quantified. After a single intravenous injection, the liver-tropic FX12m LNP formulation produced editing in approximately 37% of total liver cells. In a separate disease-relevant proof-of-concept experiment in wild-type mice, the same platform targeted the *Pcsk9* gene and achieved approximately 31% indel formation in the liver. Because inhibition of PCSK9 lowers circulating cholesterol, this approach is potentially relevant to hypercholesterolemia, including familial hypercholesterolemia; however, the study did not use a hypercholesterolemic disease model or demonstrate phenotypic correction [16].

Collectively, these studies indicate that CRISPR-Cas9 technology has achieved encouraging initial success in the treatment of inherited liver diseases. It enables precise in vivo correction of pathogenic genes and leads to long-term, stable metabolic improvement or even disease reversal in animal models. Nevertheless, with the advancement of research, the limitations of this technology have become increasingly apparent. CRISPR-Cas9 functions by cutting double-stranded DNA, and the resulting DNA double-strand breaks activate the cell’s own double-strand break repair mechanisms, such as non-homologous end joining. This endogenous repair process is highly error-prone and often introduces unintended mutations (e.g., insertions, deletions, inversions, copy number variations) [17,18], which may increase the risk of cancer. Furthermore, the relatively high off-target risk of CRISPR-Cas9 represents a safety concern that cannot be ignored in clinical translation [19]. For example, SpCas9 can still cleave even when there are mismatches between the sgRNA and the target DNA at various positions, leading to binding and editing of sequences that are not perfectly complementary to the intended target. This results in off-target effects that substantially reduce the precision of gene editing [20]. To circumvent the risks and uncertainties associated with DNA double-strand breaks, a more precise gene editing technology—base editing—has emerged. Base editing does not rely on DNA double-strand breaks or exogenous donor templates; instead, it fuses dCas9 with a deaminase to achieve direct, precise conversion of a single base in DNA or RNA. In principle, this technology avoids random mutations and reduces off-target risk while enabling efficient repair of specific point mutations, thereby providing a safer and more precise new tool for the treatment of inherited liver diseases.

## 3. Base Editing

### 3.1. Structure and Mechanism of Base Editing

**Figure 2:**
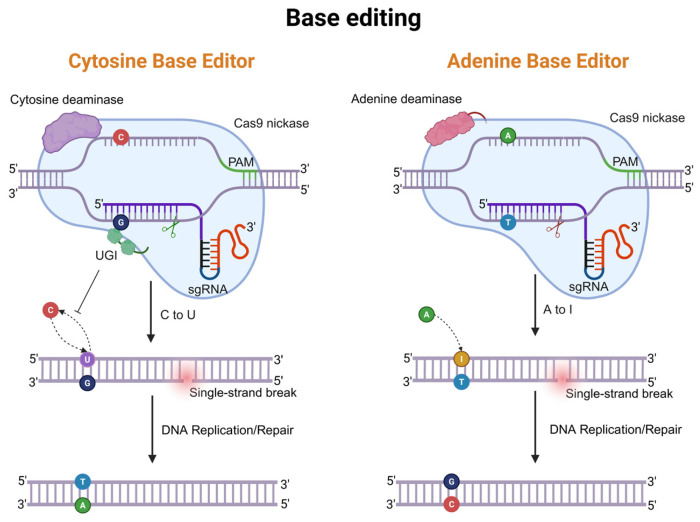
Mechanisms of cytosine and adenine base editing. In the cytosine base editor, cytidine deaminase converts C to U, UGI protects the U•G intermediate, and nCas9 creates a single-strand nick in the opposite strand; DNA replication or repair ultimately converts C•G to T•A. In the adenine base editor, an engineered adenine deaminase converts A to I, which is recognized as G during replication or repair, thereby converting A•T to G•C.

Given the issues associated with CRISPR-Cas9 technology, including DNA double-strand breaks and high indel rates, scientists have sought to develop more precise methods for gene editing. The David Liu team developed a CRISPR-Cas9-based approach known as base editing [21,22]. Base editing technologies are divided into cytosine base editors (CBEs) and adenine base editors (ABEs) [23,24]. Under the guidance of an sgRNA, the editor binds to a PAM-adjacent target, and nCas9 introduces a single-strand nick in the strand opposite the edited base. As illustrated in Figure 2, in CBE, a cytidine deaminase binds to nCas9 and, guided by an sgRNA, localizes to the target genomic DNA. The deaminase opens the DNA strand and deaminates cytosine (C) to uracil (U). Uracil glycosylase inhibitor (UGI) inhibits the removal of U and helps preserve the U•G intermediate. Through subsequent DNA replication or repair, the U is converted to thymine (T), ultimately achieving the direct substitution of a C•G base pair to a T•A base pair [25]. The challenge for ABE was that no DNA adenine deaminase exists in nature. The David Liu team used the E. coli-derived TadA protein as a starting substrate and designed an antibiotic selection scheme for directed evolution, yielding an engineered adenine deaminase [25]. This deaminase binds to DNA and converts adenine (A) to inosine (I). Inosine is recognized as guanine (G) during DNA replication and repair, ultimately achieving the conversion of an A•T base pair to a G•C base pair.

### 3.2. Progress of Base Editing in the Treatment of Inherited Liver Diseases

In principle, base editing technology has the potential to treat approximately 30% of human genetic diseases [26]. In recent years, this technology has been widely used in preclinical studies of various inherited liver diseases and has achieved significant progress.

In vivo editing therapy for phenylketonuria (PKU): Lukas Villiger et al. conducted a systematic study in the *Pah^enu2^* mouse model of phenylketonuria. They used a lipid nanoparticle (LNP) delivery system to encapsulate chemically modified SaKKH-CBE3 mRNA (5-methoxyuridine-modified) and its corresponding sgRNA into LNPs, which were then injected via the tail vein to achieve transient gene editing in the liver. Control mice received empty LNPs. In the experimental group, a single dose of 3 mg/kg achieved an editing efficiency of approximately 10.7%. Two injections spaced one week apart increased the editing efficiency to approximately 18.8% (approximately 21% in isolated hepatocytes). After treatment, the correct amino acid sequence of the mouse Phenylalanine Hydroxylase (PAH) was restored, blood phenylalanine levels decreased significantly below the therapeutic threshold of 360 µmol/L, and the coat color of the mice changed from light to dark, approaching the coat color of normal wild-type mice. This study confirmed that base editing technology functions as expected in vivo and supports the feasibility of cytidine base editing for the treatment of inherited liver diseases [27].

Exploration of therapy for hereditary tyrosinemia type 1 (HT1): Hereditary tyrosinemia type 1 is a severe metabolic disease caused by a deficiency of fumarylacetoacetate hydrolase (FAH). Early work by M. Grompe et al. attempted to introduce the full-length Fah complementary DNA via retroviral vectors into the livers of HT1 (*Fah^−/−^*) model mice. However, viral vector-mediated transgene integration carries a risk of insertional mutagenesis, and transgene expression is not subject to endogenous regulation [28,29]. Subsequently, the scientific community turned its attention to the safer, double-strand-break-free base editing technology and has achieved a series of advances:

(1) In utero gene editing: In 2018, Rossidis, Peranteau, Musunuru, and colleagues reported the first demonstration of in utero base editing for the treatment of congenital metabolic diseases. Using an adenoviral vector to deliver the BE3 base editor, the authors performed prenatal gene editing in fetal mice and evaluated two distinct therapeutic applications. In the first application, they targeted the *Pcsk9* gene—which is associated with familial hypercholesterolemia—in wild-type fetal mice. This in utero editing achieved hepatocyte editing efficiencies of 10–15%, which remained stable for up to three months after birth, and resulted in significant reductions in plasma PCSK9 protein and cholesterol levels. In the second application, they targeted the *4-hydroxyphenylpyruvate dioxygenase* (*Hpd*) gene in a murine model of hereditary tyrosinemia type 1 (HT1). Following in utero editing, *Hpd* editing efficiency increased to approximately 40% after birth, successfully rescuing the lethal phenotype in 89% of mice and restoring normal liver function. Notably, no obvious off-target effects were detected in either application. This study was the first to demonstrate the feasibility and long-term efficacy of in utero base editing for the treatment of congenital metabolic diseases [30].
(2) Strategy of creating a new start codon: In 2020, Yang, L. et al. [31] used cytosine base editing to create a novel start codon to ameliorate inherited metabolic liver disease. They generated a new HT1 mouse model containing a start codon mutation in the *Fah* gene using an adenine base editor. By targeting an upstream sequence, they created a de novo in-frame start codon to initiate FAH translation. As a result, nearly all C-to-T conversions generated a start codon and restored FAH expression, efficiently ameliorating the disease without causing off-target mutations. This study proposed that base editing-mediated creation of de novo functional elements could serve as a new strategy for treating genetic diseases [31].
(3) Ex vivo cell therapy using genetically modified hepatocyte-derived cells: In 2021, Kim, Y. et al. [32] used a chemical compound to reprogram hepatocytes from HT1 mice into expandable mouse hepatocyte-derived cells (mCdHs). They then successfully corrected the pathogenic mutation using adenine base editors (ABEs). The ABE-corrected CdHs were able to re-colonize the liver and generate FAH-positive cells, significantly improving the survival rate of HT1 mutant mice. This study demonstrated that precise gene editing in transplantable cell populations holds therapeutic potential for inherited liver diseases [32].

Base editing therapy for other inherited liver diseases:

(1) Hereditary hemochromatosis (HH): In 2022, Rovai, A. et al. [33] used an AAV8 split-vector system to deliver the adenine base editor ABE7.10 and successfully corrected the homozygous C282Y (c.845G>A) pathogenic mutation in the *Homeostatic Iron Regulator* (*Hfe*) gene of a mouse model of hereditary hemochromatosis. This G-to-A transition disrupts an intrachain disulfide bond, causing misfolding of the HFE protein and its absence at the cell membrane, which leads to systemic iron overload. ABE7.10 converts the mutant A back to G, restoring the wild-type C282 codon. After four months of low-dose injection, the editing efficiency in whole-liver DNA was approximately 6.5%. Four months after high-dose injection, the editing efficiency increased to 10.7% ± 1.2% in whole-liver DNA, 12% ± 3.6% in hepatocyte DNA, and 19.3% ± 2.2% in hepatocyte RNA. After editing, liver iron accumulation was significantly reduced, and iron metabolism parameters, including serum ferritin saturation, unsaturated iron-binding capacity, and hepcidin levels, were improved. No obvious off-target editing events were detected [33].
(2) Alpha-1 antitrypsin deficiency (AATD): In 2025, Kim, M. et al. [34] combined base editing technology with dual-organ targeting lipid nanoparticles (Dual SORT LNPs). Using the ABE8e-NGC editor, they corrected adenine (7A) to guanine (7G) at the seventh position of the *SERPINA1* gene. In a mouse model of AATD, gene correction efficiency in hepatocytes reached 30–45%, the level of mutant protein in the blood was reduced by more than 80%, and liver injury was significantly reversed [34].
(3) Carbamoyl-phosphate synthetase 1 deficiency (CPS1D): In a human study reported in 2025, Musunuru, K. et al. [35] developed a customized adenine base editor, k-ABE, targeting the Carbamoyl Phosphate Synthetase 1 (*CPS1*) Q335X (c.1003C>T) nonsense pathogenic mutation. The editor was engineered with an NGC PAM variant to broaden its compatibility with targets. It was delivered systemically via lipid nanoparticles (LNPs) for in vivo administration to a single patient with neonatal-onset CPS1 deficiency. The patient received two intravenous infusions at approximately 7 and 8 months of age at doses of 0.1 mg/kg and 0.3 mg/kg, respectively. The therapy repaired the premature stop mutation in situ and restored full-length functional protein expression. In the 7 weeks following the initial infusion, the patient tolerated increased dietary protein intake and a halving of the nitrogen-scavenger medication (glycerol phenylbutyrate) from 10.1 mL/m^2^/day to 5 mL/m^2^/day. Blood ammonia levels were maintained within the normal range (post-treatment median 13 μmol/L, interquartile range 9–28 μmol/L), and the patient recovered from consecutive viral infections without hyperammonemic crises. Preclinical studies in a patient-specific mouse model demonstrated up to 42% whole-liver corrective editing. Off-target assessment via ONE-seq, CHANGE-seq-BE, and targeted amplicon sequencing identified minimal off-target editing only at an intronic site in *ATP7B* in HuH-7 cells, which was not detected in primary human hepatocytes and was judged not to represent a biological risk. No severe treatment-related adverse events occurred. Longer follow-up is warranted to assess long-term safety and efficacy [35].
(4) Zellweger spectrum disorder (ZSD): In 2026, Gao, X.D. et al. [36], in collaboration with Cathleen M. Lutz et al., demonstrated the therapeutic potential of adenine base editing in a mouse model of ZSD. ZSD is a severe inherited liver disease caused by biallelic loss-of-function variants in *peroxisomal biogenesis factor* (*PEX*) genes required for peroxisome biogenesis. The study targeted the *Pex1*-p.G844D (c.2531G>A) pathogenic mutation—the mouse ortholog of the human *PEX1*-p.G843D (c.2528G>A) allele, which is present in approximately 30% of individuals with ZSD. ABE8e-V106W converts the mutant A back to G, restoring the wild-type codon. The study used AAV9 to deliver the ABE8e-V106W base editor to correct this mutation. The results showed that the editing efficiency in neonatal mouse livers reached up to 60%, accumulations of very long-chain fatty acids, branched-chain fatty acids, and toxic C27 bile acid intermediates were eliminated, and the liver transcriptome and pathological structure were normalized [36].

Notably, base editing technology has entered early-stage clinical trials. In March 2026, Wan, P. et al. [37] reported in Nature Medicine the first clinical study of in vivo base editing for the treatment of familial hypercholesterolemia. The researchers used lipid nanoparticles to deliver an adenine base editor (ABE) to precisely edit the *PCSK9* gene in patient livers. This resulted in a marked reduction in PCSK9 protein levels and a reduction in serum low-density lipoprotein cholesterol (LDL-C) of more than 55%, without observable severe off-target effects or toxicity [37]. It is worth noting that PCSK9 is a well-validated therapeutic target for lowering LDL-C. In 2021, the U.S. Food and Drug Administration approved inclisiran (Leqvio^®^), a small interfering RNA (siRNA) that also targets hepatic PCSK9 synthesis via RNA interference, for the treatment of adults with hypercholesterolemia, including heterozygous familial hypercholesterolemia [38]. Inclisiran has since been authorized by multiple regulatory agencies, including the European Medicines Agency (EMA) and China’s National Medical Products Administration (NMPA), for clinical use in severe hypercholesterolemia. The clinical success of inclisiran [39,40]—achieving approximately 50% LDL-C reduction with twice-yearly subcutaneous administration—underscores the clinical relevance of PCSK9 as a target and provides a valuable benchmark for evaluating the emerging base editing approach. BEAM-302 (ClinicalTrials.gov: NCT06389877) and YOLT-202 (ClinicalTrials.gov: NCT07193615) are LNP-delivered adenine base editors that correct the PiZ (c.1096G>A) mutation in *SERPINA1* for alpha-1 antitrypsin deficiency (AATD). Both formulations have been approved by the U.S. FDA. In a Phase 1/2 trial, a single 60 mg dose achieved a mean steady-state total AAT of 16.1 µM, with corrected M-AAT comprising 94% of circulating AAT and mutant Z-AAT reduced by 84% from baseline [41]. Meanwhile, YOLT-202 in a Phase 1/1a trial showed 54–57% DNA correction in liver biopsy of a 66-year-old patient, with no bystander or off-target effects, confirming efficient hepatic base editing in humans [42].

Comparison and transition to prime editing: Compared to CRISPR-Cas9 editing technology, which relies on DNA double-strand breaks to achieve gene knockout or knock-in, base editing converts one base pair directly into another at the target site without cutting the DNA double helix. This greatly reduces the risk of indel mutations, chromosomal translocations, and other genomic instabilities caused by DNA break repair, significantly improves the safety of gene editing, and has shown remarkable therapeutic potential in various preclinical studies of inherited liver diseases. However, base editing is primarily suitable for correcting mutations caused by base substitutions (C•G to T•A or A•T to G•C). It generally cannot repair disease-causing alleles that result from insertions, deletions, or more complex base transversions, which account for a substantial proportion of genetic variants. Moreover, when the activity window of a base editor (BE) encompasses multiple editable bases, unintended bystander mutations may occur [23,24,43], limiting its precise application to certain sequences. In this context, the David R. Liu team developed prime editing (PE) technology in 2019. Also based on the CRISPR-Cas9 system, prime editing enables any base substitution, small insertions or deletions, and even targeted insertion or deletion of larger sequences without introducing DNA double-strand breaks. Compared with base editing, prime editing has a much broader editing scope and is therefore described as a search-and-replace precision gene editing tool [44]. This technology possesses an extremely wide editing spectrum, allowing precise manipulation of the human genome. It has the potential to correct up to 89% of known human genetic diseases, offering a more versatile intervention strategy for many refractory diseases, including inherited liver diseases.

## 4. Prime Editing

### 4.1. Structure and Mechanism of Prime Editing

The prime editor consists of a fusion enzyme and a prime editing guide RNA (pegRNA). The fusion enzyme consists of a reverse transcriptase derived from M-MLV and SpCas9. The M-MLV-derived reverse transcriptase retains its reverse transcription activity, while the SpCas9 protein is a modified version of the native Cas9 enzyme. Native Cas9 possesses two nuclease activities that cleave both strands of DNA; after modification, it retains the activity to cleave only one strand [45]. The pegRNA plays a critical role in the prime editing system. The 5′ region of the pegRNA contains the guide sequence, which is complementary to the target DNA and directs Cas9 binding to the target locus. Immediately downstream of this guide sequence lies a scaffold sequence that recruits the Cas9 protein, forming the functional prime editing complex. The 3′ end includes two key functional elements: a primer-binding site (PBS) and a reverse transcription template (RT template). After Cas9 creates a single-strand nick in the target DNA, the PBS sequence hybridizes with the nicked DNA strand, serving as a primer for reverse transcription. The reverse transcriptase then uses the RT template to synthesize a new DNA strand containing the desired edit [46].

**Figure 3:**
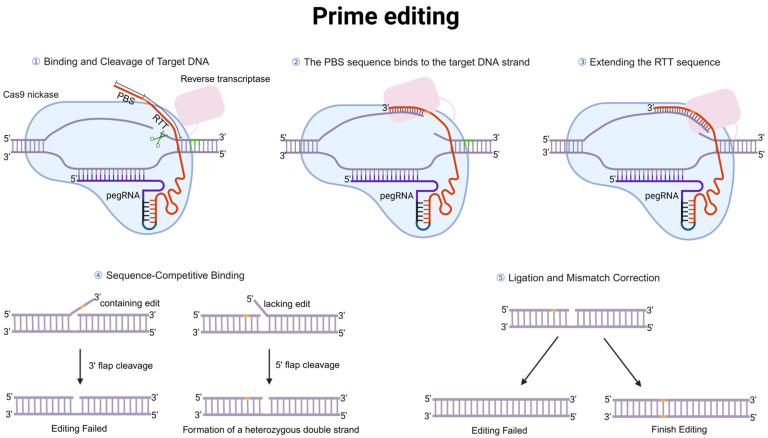
Mechanism of prime editing. The pegRNA-programmed SpCas9(H840A)-reverse transcriptase complex nicks the target DNA. The PBS anneals to the exposed 3′ end, and the RT template is copied to form an edited 3′ flap. Competition between the edited 3′ flap and the unedited 5′ flap determines the outcome: cleavage of the 3′ flap results in editing failure, whereas cleavage of the 5′ flap followed by ligation forms a heteroduplex that is resolved by mismatch repair or DNA replication to either retain the programmed edit or restore the original sequence.

The working mechanism of prime editing (PE) is illustrated in Figure 3. First, the pegRNA binds to the editor protein (a fusion of SpCas9(H840A) and M-MLV reverse transcriptase) to form a complex. Guided by the pegRNA, the complex localizes to the target DNA site, and SpCas9 introduces a single-strand nick at the third base upstream of the PAM sequence (NGG), exposing a 3′ DNA end. Subsequently, the PBS sequence of the pegRNA hybridizes with the nicked single-stranded DNA, and the reverse transcriptase uses the RT template to synthesize a new DNA strand containing the desired edit. This process generates a 3′ DNA flap carrying the edited sequence at the target site, which competes with the original unedited 5′ flap for hybridization with the complementary strand. Cleavage of the edited 3′ flap leads to editing failure, whereas cleavage of the unedited 5′ flap followed by ligation forms an edited/unedited heteroduplex. After cellular DNA mismatch repair or DNA replication machinery recognizes the heteroduplex, the edited sequence may be retained; conversely, if repair favors the unedited strand, the original sequence is restored. Through this search-and-replace mechanism, the PE system achieves precise sequence writing without relying on DNA double-strand breaks [44].

### 4.2. Progress of Prime Editing in the Treatment of Inherited Liver Diseases

Since emerging in 2019, prime editing, a precise gene editing technology that does not rely on DNA double-strand breaks, has shown great promise for treating inherited liver diseases. Its unique working mechanism—achieving precise sequence writing through two-step hybridization of the pegRNA’s primer-binding site (PBS) and reverse transcription template (RT template) with the target DNA—confers an extremely low off-target risk [47,48], which is particularly important for hepatocyte gene therapy that requires lifelong stable expression. This low off-target profile was detailed in a publication by David R. Liu et al. [44]. Using HEK293T cells as a model, they examined known high off-target sites for Cas9 in the *HEK3*, *HEK4*, *EMX1*, and *FANCF* genes. The results showed that prime editing produced off-target frequencies of <0.1%, <2.2 ± 5.2%, <0.1%, and <0.13 ± 0.11% at these four sites, respectively. In contrast, conventional Cas9-sgRNA exhibited off-target frequencies as high as 16 ± 16%, 60 ± 26%, 48 ± 28%, and 4.3 ± 5.6% at the same sites. The molecular mechanism underlying this marked difference involves prime editing with two additional DNA hybridization steps: hybridization of the target DNA with the PBS, and hybridization of the target DNA with the RT template. Because off-target sites differ in nucleotide sequence from the on-target site, these differences make effective hybridization difficult. Efficient hybridization is a prerequisite for successful editing, and the low hybridization rate at off-target sites directly results in very low editing at those sites, thereby manifesting as a globally low off-target effect (i.e., high specificity) of the prime editing system [44]. This characteristic establishes an important safety foundation for the application of prime editing in inherited liver diseases.

Since 2021, multiple studies have successively validated the therapeutic potential of prime editing in various animal models of inherited liver diseases.

(1) Alpha-1 antitrypsin deficiency (AATD): In 2021, Liu, P. et al. [49] optimized the nuclear localization signal (NLS) of the prime editor PE2 and achieved in vivo correction of the pathogenic mutation in adult mouse livers. Using PE2, they successfully corrected the *SERPINA1* E342K mutation in AATD model mice and demonstrated that this system could be used to generate liver cancer models. Furthermore, the team delivered the prime editor using a split intein-based dual AAV8 system, achieving sustained editing in mouse livers. This study provided an important proof-of-concept for prime editing-based therapy of inherited liver diseases [49].
(2) Hereditary tyrosinemia type 1 (HT1): In 2021, Jang, H. et al. [50] evaluated the therapeutic potential of prime editing in adult *Fah^mut/mut^* mouse models of tyrosinemia type 1. Through high-throughput screening, they identified efficient pegRNAs and delivered the PE3 system via hydrodynamic injection. PE3 achieved an average of 11.5% targeted gene correction in the liver, restored normal splicing of exon 8, resulted in FAH protein expression in approximately 61% of hepatocytes, and significantly improved mouse survival. Notably, compared to conventional Cas9-mediated HDR, PE3 induced very low indel frequencies (average 0.78%), highlighting its advantage in precise repair [50]. In the same year, Wen Xue et al. further developed a prime editing-based deletion and repair method (PEDAR). Using a pair of pegRNAs, they deleted an ~1.38 kb pathogenic insertion in exon 5 of the *Fah* gene in HT1 mouse models while simultaneously inserting a 19 bp corrective sequence to restore the reading frame. Following hydrodynamic delivery, FAH-positive hepatocytes accounted for 0.76% in the initial liver. After NTBC withdrawal, corrected hepatocytes proliferated and repopulated the liver, and deep sequencing revealed an accurate repair efficiency of 78.2%, successfully restoring FAH expression and normal hepatocyte morphology [51].
(3) Phenylketonuria (PKU): In 2022, Böck et al. [52] designed a compact SpCas9 prime editor lacking the RNase H domain (PE2^ΔRnH^) to fit within the packaging capacity of AAV vectors. At the *Dnmt1* locus, AAV8 delivery achieved 14.4% editing efficiency in neonatal mice, while adenoviral (AdV) delivery significantly increased efficiency to 58.2%. AdV delivery of the compact prime editor corrected the pathogenic *Pah^enu2^* mutation in a PKU mouse model, with an average correction efficiency of 11.1% in neonatal mice, leading to a therapeutic reduction in blood phenylalanine levels without detectable off-target mutations or long-term liver inflammation [52]. In 2025, Rothgangl et al. [53] developed two transient prime editing strategies for the liver: a “dual-system” approach using AAV for stable pegRNA expression combined with LNP delivery of PE-mRNA, and an “all-LNP” approach co-delivering PE-mRNA and synthetic pegRNA via LNPs. In the PKU mouse model (*Pah^enu2^*), after three doses, the dual-system achieved 20.7% editing efficiency, reducing blood phenylalanine levels below the therapeutic threshold. Co-delivery of PE7-mRNA and chemically modified pegRNA using LNPs achieved 8.0% editing efficiency at the *Pah^enu2^* locus, reaching the threshold for therapeutic correction. Neither strategy caused detectable off-target editing or significant liver toxicity [53].
(4) Familial hypercholesterolemia (FH): In 2023, Davis, J.R. et al. [54] developed an optimized dual-AAV prime editing system based on SpCas9 and a truncated reverse transcriptase lacking the RNase H domain. Using AAV9 delivery, they successfully installed the coronary artery disease-protective *Pcsk9* Q155H mutation in adult mouse livers. Eight weeks after injection, the average editing efficiency in the liver reached 39%, resulting in sustained reductions of approximately 20% in plasma total cholesterol and 27% in low-density lipoprotein cholesterol. CIRCLE-seq detected no off-target editing at predicted sites, and liver enzyme and histological analyses revealed no overt toxicity [54]. While the PCSK9 target has been clinically validated by the approved siRNA therapeutic inclisiran, the prime editing approach offers a fundamentally different strategy, permanent DNA-level correction versus transient RNA silencing, with the potential for durable, one-time efficacy. However, the transition from preclinical proof-of-concept in mice to human application will require overcoming challenges associated with dual-AAV delivery, including vector immunogenicity, packaging efficiency, and long-term safety in the liver.

Prime editing has now advanced from preclinical proof-of-concept to clinical-stage development for inherited liver diseases. Prime Medicine’s PM577a is an LNP-delivered prime editor that targets the H1069Q mutation in the *ATP7B* gene, which accounts for approximately 30–50% of Wilson disease-associated variants in the United States and Europe. In June 2026, the therapy was approved by New Zealand’s Medsafe, becoming the first in vivo gene editing therapy to receive clinical authorization. The Phase 1/2 study will assess safety, copper efflux, ceruloplasmin, and hepatic copper, with initial data expected in 2027 [55].

In summary, as inherited liver diseases are often caused by mutations in a single gene, traditional therapies such as medication and liver transplantation cannot fundamentally resolve the problem, and transplantation faces challenges, including donor shortages and immune rejection. Gene editing technology enables precise correction of pathogenic mutations directly at the genome level, providing a revolutionary therapeutic strategy for inherited liver diseases that are difficult to treat with conventional approaches. Among these technologies, CRISPR-Cas9, as a pioneering tool, achieves gene editing by inducing DNA double-strand breaks and harnessing cellular repair mechanisms; however, its potential off-target effects and repair uncertainty limit clinical application. To improve safety, base editing technology emerged, which achieves efficient single-base conversion without introducing DNA double-strand breaks, thereby greatly reducing adverse reactions. The latest prime editing technology further expands editing capabilities and can theoretically perform all types of base substitutions, as well as small insertions and deletions, earning it the reputation of a “gene word processor” with extremely high precision.

To systematically illustrate the progress of these technologies in the treatment of inherited liver diseases, Table 1 summarizes key case studies.

## 5. Challenges and Future Perspectives of Gene Editing Technologies for Inherited Liver Diseases

The rapid development of gene editing technologies has brought hope for curative treatment of inherited liver diseases, but many challenges remain. From editing efficiency and off-target effects to in vivo delivery and clinical translation, each step requires substantial breakthroughs by researchers.

First, different gene editing technologies each have their own efficiency limitations when applied to liver disease treatment. CRISPR-Cas9 relies on DNA double-strand breaks and endogenous repair mechanisms; although it can achieve gene knockout, homology-directed repair is extremely inefficient in non-dividing hepatocytes, and unintended insertions and deletions (indels) are frequently introduced. Base editing does not induce DNA double-strand breaks, but it is only applicable to specific base conversions, and “bystander” mutations within its activity window may compromise precision. Prime editing, the newest and most promising tool, involves the coordinated action of multiple components and regulation by endogenous repair or replication factors, resulting in generally low editing efficiency. Currently reported editing efficiencies in the liver are mostly in the range of 10–20% [32,45,46,47]. For metabolic diseases that require a high percentage of corrected cells to reach the therapeutic threshold, this level of efficiency still needs to be improved to meet clinical demands. Meanwhile, protein engineering and RNA engineering offer hope for future efficiency enhancements. For prime editing, strategies such as truncating the reverse transcriptase (PE2^ΔRnH^) to meet delivery requirements [45,46], fusing RNA-binding proteins to enhance pegRNA stability (PE7) [47], and artificial intelligence-assisted pegRNA optimization may further improve editing efficiency. In addition, emerging CRISPR-based technologies such as programmable addition via site-specific targeting elements (PASTE), which combines prime editing with integrase-mediated large-gene insertion, hold considerable promise for the treatment of hereditary liver diseases. Although current evidence is limited to proof-of-concept studies demonstrating gene integration in human hepatocytes and mouse liver [57], and no peer-reviewed therapeutic application for a specific liver disease has been reported to date, the potential for correcting large-gene defects in the liver is evident. Future improvements in delivery efficiency, on-target integration rates, and safety profiles will be critical to translate this technology into clinical therapies for genetic liver disorders.

Second, in vivo delivery of gene editing tools is a core issue for future clinical translation. Adeno-associated virus (AAV), with its advantages of precise delivery, high safety, and durable efficacy, has become the leading vector for treating rare genetic diseases and has been approved for in vivo gene therapy and gene editing applications [58,59]. However, using AAV for prime editing still faces major challenges. Although prime editing has shown great potential in cultured cells and in vitro models since its inception in 2019, its safety and efficacy in vivo have not been fully validated. Viral vectors may cause off-target effects and elicit immune responses, limiting clinical application, and there is a need to develop safe, efficient, and tissue-permissive delivery systems [60]. Furthermore, the coding sequence of the prime editing system (~6.3 kb) exceeds the packaging limit of commonly used AAV vectors (~4.7 kb) [61,62]. Although several research teams, including the one of David Liu, have successfully split the PE system into two parts for delivery via dual AAV vectors, thereby overcoming the packaging capacity limitation [63,64,65], the reported editing efficiencies of dual-AAV strategies are only 1.7–13.5%, far below the therapeutic threshold, these approaches are restricted to a few target organs/tissues such as the liver and retina [49,52,66,67]. Finally, long-term stability of the editing products after prime editing-based treatment of liver diseases, potential cytotoxicity, and effects on the liver microenvironment have not been thoroughly investigated, and more studies are needed to evaluate long-term safety and efficacy.

Non-viral delivery methods represent an important alternative for delivering gene editing systems [68,69]. They mainly include physical and chemical approaches. Physical methods, such as microinjection, electroporation, and hydrodynamic injection, achieve transfection of biomacromolecules by disrupting or altering the cell membrane. Electroporation is the most widely used in vitro transfection technique [32,70,71,72]. Although hydrodynamic injection can efficiently target multiple organs [50,51], it may cause severe acute side effects, posing challenges for clinical translation [70]. Chemical methods utilize nanoparticles for delivery, with the advantage that gene editing tools have a short residence time in vivo, which helps reduce off-target risk and immunogenicity [73,74]. However, the application of such chemical materials for delivering the prime editor system remains unknown and urgently needs investigation.

Beyond technical challenges, clinical translation also requires systematic consideration of the specific disease type, patient developmental stage, and the overall regulatory network of hepatic metabolism. Building on experience in gene therapy for inherited liver diseases, research on gene therapy for conditions such as Crigler–Najjar syndrome (CNS) and progressive familial intrahepatic cholestasis (PFIC) has shown that while AAV-mediated gene replacement therapy can achieve significant reductions in bilirubin levels in young animals, repeated dosing is hindered by the formation of neutralizing antibodies. Combining immunomodulators (such as rapamycin) can prolong transgene expression [75,76,77]. These studies suggest that restricting the editing window to the neonatal or early developmental stage, together with immunomodulatory regimens, may achieve durable correction while avoiding repeated dosing. Furthermore, a long-term metabolic study in *Ugt1a1^−/−^* mice revealed that bilirubin metabolic disorder can trigger compensatory changes in the redox state and overall metabolic network [78]. This indicates that future gene editing therapies should not be limited to repairing a single gene but should incorporate multi-omics analysis to assess global remodeling of liver metabolic pathways after editing, and when necessary, combine metabolic regulation or anti-inflammatory interventions to achieve synergistic treatment.

Based on the above analysis, we believe that the future development of gene editing technologies for inherited liver diseases should focus on the following directions. First, develop delivery systems that combine high liver targeting efficiency with low immunogenicity, particularly strategies that enable lifelong cure with a single administration in neonates or infants. Although dual-AAV delivery of large editing tools such as the prime editor has overcome packaging limitations, its editing efficiency remains far below the therapeutic threshold required for diseases that demand correction in a high proportion of hepatocytes (e.g., Crigler–Najjar syndrome). Optimizing the ratio of dual AAVs, screening efficient promoters, and improving hepatocyte transduction efficiency could help address this issue. Second, explore combination strategies of gene editing with immune modulation and metabolic intervention. Immunomodulation can effectively prolong transgene expression [76], and this concept can be extended to CRISPR-Cas9 editing systems to reduce immune responses triggered by the Cas9 protein and viral capsids, thereby enhancing the durability of editing efficiency. Third, promote early-intervention studies of gene editing technologies in rare pediatric diseases. Inherited liver diseases often present severe phenotypes in the neonatal or infantile period [75,76]. The optimal timing of early intervention, administration routes, and safety windows are scientific questions that urgently need to be clarified for future clinical translation. Fourth, to effectively improve the precision and safety of gene editing, artificial intelligence can be used to optimize sgRNA design, predict off-target effects, and, together with tools such as AlphaFold, mine and engineer novel Cas enzymes, providing important support for the clinical translation of liver-targeted therapies.

In summary, CRISPR-Cas9-based gene editing technologies offer the potential to correct the root cause of inherited liver diseases. However, their successful clinical translation will require systematic breakthroughs in delivery systems, editing efficiency, long-term safety, and combination treatment strategies. Future efforts should continue to advance AAV-based liver gene therapy, disease model generation, and metabolic mechanism studies, focusing on the key scientific issues outlined above. Pushing gene editing technologies from basic research to clinical translation will provide new solutions for the curative treatment of inherited liver diseases.

## Figures and Tables

**Table 1:** Overview of the application of gene editing technology in inherited liver diseases.

Disease	Editing System	Editing Strategy	Delivery Method	Outcome	Reference
Ornithine transcarbamylase deficiency (OTCD)	CRISPR-Cas9	Correction of G>A mutation in OTC gene	Dual AAV8	Repair of 10% of hepatocyte mutations	[13]
Alpha-1 antitrypsin deficiency (AATD)	CRISPR-Cas9	Correction of G>A mutation in exon 5 of *Serpina1*	Dual AAV	14–18% of hepatocytes express corrected M-AAT	[14]
Hereditary tyrosinemia type 1 (HT1)	CRISPR-Cas9	Correction of loss-of-function variant in *Fah* gene	Hydrodynamic injection (HTVI)	Proportion of FAH+ hepatocytes increased to 33.5%	[12]
Familial hypercholesterolemia (FH)	CRISPR-Cas9	Correction of nonsense mutation in LDLR	AAV	LDLR protein restored to ~18% of wild-type levels; ~20% of hepatocytes re-express LDLR	[15]
Phenylketonuria (PKU)	CBE3	Correction of T>C mutation in *PAH*	LNP	Restoration of correct PAH amino acid sequence; blood phenylalanine decreased below therapeutic threshold (360 µmol/L)	[27]
Familial hypercholesterolemia (FH)	BE3	Introduction of stop codon in *Pcsk9* (W159)	Adenovirus (AdV)	10–15% allele editing efficiency in hepatocytes	[30]
Hereditary hemochromatosis (HH)	ABE7.10	Correction of G>A mutation in *HFE*	Dual AAV8	Significant reduction in liver iron accumulation; improvement in ferritin saturation, UIBC, and hepcidin levels	[33]
Alpha-1 antitrypsin deficiency (AATD)	ABE8e	Correction of G>A mutation in *SERPINA1*	LNP	30–45% gene correction efficiency in hepatocytes; >80% reduction in mutant protein; reversal of liver injury	[34]
Hereditary tyrosinemia type 1 (HT1)	ABE6.3	Correction of G>A mutation in *Fah*	LNP	Restoration of functional FAH expression in ~1% of hepatocytes	[56]
Carbamoyl-phosphate synthetase 1 deficiency (CPS1D)	k-ABE	In situ repair of premature stop mutation	LNP	Excellent targeted correction in patient liver; reversal of hyperammonemia; long-term normal blood ammonia; improved protein tolerance	[35]
Zellweger spectrum disorder (ZSD)	ABE8e-V106W	Correction of Pex1-p.G844D pathogenic mutation	AAV9	Up to 60% editing efficiency in neonatal mouse liver; elimination of VLCFA, branched-chain fatty acids, and toxic bile acid intermediates; normalization of liver transcriptome and pathology	[36]
Familial hypercholesterolemia (FH)	ABE	Correction of G>C mutation in *Pcsk9*	LNP	The patient showed a marked reduction in PCSK9 protein levels, and serum low-density lipoprotein cholesterol (LDL-C) decreased by more than 55%.	[37]
Familial hypercholesterolemia (FH)	PE3	Correction of G>C mutation in *Pcsk9*	Dual AAV9	Average 39% editing efficiency at 8 weeks post-injection	[54]
Phenylketonuria (PKU)	PE2ΔRnH	Correction of T>C mutation in exon 7 of *Pah*	AAV/AdV	11.1% editing efficiency; phenylalanine levels reduced to 100 ± 34 µM	[52]
Phenylketonuria (PKU)	PE7	Correction of T>C mutation in exon 7 of *Pah*	AAV9/LNP	20.7% editing efficiency after three doses; blood phenylalanine below therapeutic threshold	[53]
Alpha-1 antitrypsin deficiency (AATD)	PE2	Correction of G>A mutation in exon 5 of *Serpina1*	Dual AAV8	6.7% editing efficiency	[49]
Hereditary tyrosinemia type 1 (HT1)	PE3	Correction of G>A mutation in *Fah*	Hydrodynamic tail vein injection	Average 61% FAH+ hepatocytes at day 40	[50]
Hereditary tyrosinemia type 1 (HT1)	PEDAR	Deletion of 1.38 kb pathogenic insertion in exon 5 of *Fah* insertion of 19 bp correct sequence	Hydrodynamic tail vein injection	Initial FAH+ hepatocytes 0.76%; after NTBC withdrawal, corrected hepatocytes repopulate liver; deep sequencing shows 78.2% precise repair	[51]

## Data Availability

No new data were created or analyzed in this study. Data sharing does not apply to this article.
